# Evaluation of a Mathematical Model of Rat Body Weight Regulation in Application to Caloric Restriction and Drug Treatment Studies

**DOI:** 10.1371/journal.pone.0155674

**Published:** 2016-05-26

**Authors:** Jangir Selimkhanov, W. Clayton Thompson, Terrell A. Patterson, John R. Hadcock, Dennis O. Scott, Tristan S. Maurer, Cynthia J. Musante

**Affiliations:** 1 Cardiovascular and Metabolic Diseases Research Unit, Pfizer Worldwide Research and Development, Cambridge, Massachusetts, United States of America; 2 Pharmacokinetics, Dynamics and Metabolism, Pfizer Worldwide Research and Development, Cambridge, Massachusetts, United States of America; Bilkent University, TURKEY

## Abstract

The purpose of this work is to develop a mathematical model of energy balance and body weight regulation that can predict species-specific response to common pre-clinical interventions. To this end, we evaluate the ability of a previously published mathematical model of mouse metabolism to describe changes in body weight and body composition in rats in response to two short-term interventions. First, we adapt the model to describe body weight and composition changes in Sprague-Dawley rats by fitting to data previously collected from a 26-day caloric restriction study. The calibrated model is subsequently used to describe changes in rat body weight and composition in a 23-day cannabinoid receptor 1 antagonist (CB1Ra) study. While the model describes body weight data well, it fails to replicate body composition changes with CB1Ra treatment. Evaluation of a key model assumption about deposition of fat and fat-free masses shows a limitation of the model in short-term studies due to the constraint placed on the relative change in body composition components. We demonstrate that the model can be modified to overcome this limitation, and propose additional measurements to further test the proposed model predictions. These findings illustrate how mathematical models can be used to support drug discovery and development by identifying key knowledge gaps and aiding in the design of additional experiments to further our understanding of disease-relevant and species-specific physiology.

## Introduction

Obesity is a growing epidemic worldwide with significant health and economic impacts [1]. Non-pharmacologic interventions such as diet and exercise have shown only limited and mostly temporary success [2]. Concurrently, while some promising targets for obesity pharmacotherapy (e.g., [3–5]) have been identified, the pharmaceutical industry generally has struggled to translate pre-clinical results into safe and effective anti-obesity therapies in humans. Our ability to identify and understand metabolically-relevant differences between species may improve translation of pre-clinical research to clinical success.

The development and application of physiologically based mathematical models can help to identify key knowledge gaps and to generate testable hypotheses to improve our understanding of disease mechanisms. Species-specific *in silico* models for humans and rodents can help in the evaluation of how preclinical results for a novel anti-obesity target or compound will translate to human efficacy. A well-supported mathematical model of human energy metabolism has previously been developed by Hall [6]; however, for translation, similar models are required for pre-clinical animals. A number of models of murine metabolism have been developed recently to describe changes in body weight (BW) and body composition (BC) under varying conditions [7–11]. These modeling efforts have utilized a two-dimensional model of energy balance, which relates changes in fat mass (FM) and fat-free mass (FFM) to the energy imbalance between food intake (FI) and energy expenditure (EE). Guo and Hall [8] were successful in using this model to predict BW and BC changes in several long term caloric restriction studies, while Gennemark et al. [9] applied the model to describe mouse BW changes due to several pharmacotherapies. We are interested in adapting this model to another commonly used rodent model, the Sprague-Dawley (SD) rat, to evaluate how well it can capture the effects of short-term caloric restriction and pharmacotherapy studies in a different species. The ability of a model to predict the effects of pharmacotherapy as well as caloric restriction is key for its application to common study designs in obesity research that utilize pair-feeding arms.

To evaluate the model, we identified two independent historical studies that measured FI, BW, and BC in response to two different interventions in diet-induced-obese (DIO) SD rats. In one study, rats were subjected to 26-day caloric restriction (CR); in the other rats were exposed to a 23-day pharmacological treatment with rimonabant. Rimonabant is a small molecule functional antagonist of G-protein coupled cannabinoid receptor 1 (CB1Ra) developed by Sanofi-Aventis (SR 141716) [12]. CB1 receptors are predominantly expressed in the central nervous system but have also been found in GI tract, adipose tissue, and cardiovascular system [12]. Modulation of central CB1 receptors has been shown to affect rewarding aspects of food consumption, while the role of peripheral receptors remains inconclusive [12]. Clinically significant weight loss and improvements in several metabolic risk factors led to rimonabant becoming the first selective CB1Ra to be approved in Europe ten years ago [12]. However, due to the increase in depression, anxiety, and other psychiatric adverse events among people taking rimonabant, the drug was never approved by the US Food and Drug Administration and subsequently removed from the European markets [12, 13]. Repeated oral administration of rimonabant has been demonstrated to cause dose-dependent BW loss in DIO rodent models due to reduced FI and increased EE [14–18].

In this work we find that the adapted two-dimensional model is unable to capture CR- and CB1Ra-induced FM and FFM changes simultaneously, which suggests a potential limitation of the model for application to short-term studies involving body composition. Specifically, we show that a key assumption of the model that describes the relative changes in body composition components does not hold in these short-term interventional studies in SD rats. We address this shortcoming by proposing a new model that is capable of fitting the BC data. We then propose additional measurements that are needed to further evaluate this model for broader application.

## Experimental Materials and Methods

The following two rat studies were conducted independently of each other and not specifically for the analysis carried out in this work. They were chosen to capture different types and magnitudes of treatment and study length that are common in pre-clinical drug development. All procedures performed on animals in the following two studies were in accordance with established guidelines and regulations, and were reviewed and approved by the Pfizer Institutional Animal Care and Use Committee. The CB1Ra study results have previously been presented at the 2nd Asia Pacific International Society for the Study of Xenobiotics Meeting (May 11–13, 2008) [19].

### Caloric restriction study

#### Animals

Male DIO SD rats weighing 640 g (62 g STD) were obtained from Charles River Laboratories, Inc (Raleigh, NC). During this period, animals had free access to water and a 40% fat (4.55 kcal/g) diet (Test Diet 5342).

#### Body weight and food intake study

Baseline FI was established over 4 days, 12 days before the start of caloric restriction period. Five groups of rats (n = 10 per group) were provided identical diets with the control group receiving food ad libitum, and the other 4 groups calorically restricted at 10%, 20%, 30%, and 40% of baseline FI for a period of 26 days. FI and BW were measured every 24 hours during the caloric restriction phase of the study. BC was measured using Hologic dual-energy x-ray absorptiometry (DXA) 3 days prior to and 12 and 22 days after the start of caloric restriction.

### CB1Ra study

#### Animals

Male DIO SD rats weighing 695 g (75 g STD) were obtained from Charles River Laboratories, Inc (Raleigh, NC). Rats were allowed to acclimate to single housing on a 12-hour dark light cycle (lights off at 4 PM and on at 4 AM) for one week prior to the study. During this period, animals had free access to water and a powdered 45% fat diet (4.7 kcal/g) (Purina Western Diet 1810104).

#### Body weight and food intake study

CB1Ra, formulated in 0.5% methylcellulose, was administered to rats in 23 consecutive daily oral doses of 3, 10 and 30 mg/kg (n = 10 per group). Vehicle was administered to another group (n = 9) for 23 consecutive days. The amount of food consumed and BW were measured every 24 hours during drug treatment phase and for 15 days after cessation of drug administration (washout phase). BC was measured using DXA scanner 3 days prior to and 24 and 42 days after the start of drug administration.

#### Reagents

CB1Ra (CP 330947, PF 1867088), rimonabant HCl, was synthesized at Pfizer Global Research and Development in Groton, CT (Rinaldi-Carmona, Barth et al. 1994).

## Quantitative Methods

### Two-dimensional model of rat metabolism

To develop an energy balance model of rat metabolism that describes changes in BC, we adapted a model previously validated in mice [8]. Briefly, the model consists of 2 ordinary differential equations describing changes in fat mass (*FM*) and fat-free mass (*FFM*) that are driven by the energy imbalance caused by the difference in FI (*I*) and EE (*E*):

$$\begin{array}{cc} \rho_{FM}\frac{dFM}{dt} & =\frac{\rho_{FM}}{\rho_{FM}+\rho_{FFM}\alpha }\left(I-E\right)\\ \rho_{FFM}\frac{dFFM}{dt} & =\frac{\rho_{FFM}\alpha }{\rho_{FM}+\rho_{FFM}\alpha }\left(I-E\right). \end{array}$$ (1)

The function $\alpha =\frac{dFFM}{dFM}$ describes the longitudinal changes in FFM relative to FM which, together with the initial conditions for the model, defines a trajectory that constrains the model solution [20]. Food intake energy (*I*) is measured directly and is the only input into the model (S1 Fig). Energy expenditure (*E*) is defined by the following equation

$$\begin{array}{c} E=K+\beta \left(I-I_{0}\right)+\left(\lambda +\gamma_{FM}\right)FM+\left(\lambda +\gamma_{FFM}\right)FFM\\ \;+\eta_{FM}\frac{dFM}{dt}+\eta_{FFM}\frac{dFFM}{dt}. \end{array}$$ (2)

All model parameters are described in the following section.

### Model translation and calibration

Translation was defined as the process of identifying and updating model parameter values according to known species-specific differences, while calibration was defined as the process of fitting the remaining unknown parameters to available study data (S1 Supporting Dataset). Parameter values for energy densities (*ρ*_*FM*_, *ρ*_*FFM*_) and deposition costs (*η*_*FM*_, *η*_*FFM*_) were assumed to be species-invariant and were taken directly from [8]. Diet-induced thermogenesis (DIT) describes EE associated with diet composition and amount of food ingested. The value for the DIT parameter (*β*) was obtained from previous analysis of SD rat growth [21]. Rat metabolic rates for FM (*γ*_*FM*_) and FFM (*γ*_*FFM*_) were estimated using Kleiber’s Law as in [8], and assuming a weight of 350 g for a lean rat. The function *α* was assumed to be constant and determined by total least squares fit to BC data from the CR study (S2 Fig). Translated parameter values are shown in S1 Table.

In addition to the translated parameter values, there were two unknown EE parameters describing physical activity (*λ*) and basal thermogenesis (*K*). These parameters could not be simultaneously identified (S3 Fig). Therefore, we set the value of parameter *K*_*CR*_ (the value of *K* specific to the CR study) to zero and estimated the value for *λ* by fitting the model to CR data, with the error between the model simulations (*BW*, *FM*) and mean data measurements ($\hat{BW}$, $\hat{FM}$) calculated as the sum of mean squared errors for BW and FM

$$\begin{array}{c} Error=\frac{{\left(BW-\hat{BW}\right)}^{2}}{N_{BW}}+\frac{{\left(FM-\hat{FM}\right)}^{2}}{N_{FM}}, \end{array}$$ (3)

where *N*_*BW*_ and *N*_*FM*_ are the number of BW and FM measurements respectively. We used only BW and FM measurements for fitting since FFM was not measured directly, but calculated according to $\hat{FFM}=\hat{BW}-\hat{FM}$. This error function was used in all subsequent fits of the model. The model was simulated from the first BC to the last BC measurement in each study. Missing FI values between initial BC measurements and the start of intervention were set to the baseline FI. Similarly, missing FI values at the end of the CB1Ra study, before the last BC measurement, were set to the last measured FI value.

To fit data from the CB1Ra treatment study, we assumed that the effect of drug treatment (*d*) on EE is described by a simple dose-dependent Hill function

$$\begin{array}{c} d\left(X\right)=D_{0}+D_{\text{max}}\frac{X}{ED_{50}+X} \end{array}$$ (4)

where *X* is the drug dose, *D*_0_ is defined as handling stress that is present in all animal groups, *D*_max_ is the maximum drug effect, and *ED*_50_ is the drug dose that results in half-maximal effect. The drug effect term was applied only during the treatment phase (*T* = [0, 23]), during which the drug was administered. Furthermore, the effect of the drug on EE was assumed to be independent of other EE components: *E*(*X*) = *E* + *d*(*X*).

Due to possible differences between the two studies, such as handling stress and study location, we assumed that basal thermogenesis in the drug treatment study (*K*_*CB*1_) may take on a different value from *K*_*CR*_, while the parameter *λ* was carried forward from the initial model fit to CR data. We used simulated annealing to fit the 4 additional EE parameters (*K*_*CB*1_, *D*_0_, *D*_max_, *ED*_50_) to the CB1Ra drug treatment study data. Calibrated parameter values are shown in S2 Table.

### Derivation of the *α*-free energy balance model

In addition to the adapted two-dimensional model, we derived a new *α*-free model of rat energy balance and BC. This model was used to evaluate the EE function without the constraint imposed by the function *α*. Starting with the equations for mass and energy balance,

BW=FFM+FM (5)

$$I-E=\rho_{FM}\frac{dFM}{dt}+\rho_{FFM}\frac{dFFM}{dt}$$ (6)

we solved for changes in BC in terms of FI energy (*I*), EE (*E*), and body weight (*BW*). Differentiating Eq 5 with respect to time and plugging into Eq 6 for $\frac{dFFM}{dt}$, we obtained the following *α*-free differential equation describing changes in FM

$$\begin{array}{cc} \left(\rho_{FM}-\rho_{FFM}\right)\frac{dFM}{dt} & =I-E-\rho_{FFM}\frac{dBW}{dt}, \end{array}$$ (7)

with FFM computed using the mass balance Eq 5. Unlike the two-dimensional model, the *α*-free model requires BW as an additional input to simulate. To use BW data ($\hat{BW}$) as an input, we first fit a cubic smoothing spline (*S*_*BW*_(*t*)) to $\hat{BW}$ using MATLAB’s fit.m function with smoothing parameter of 0.2 to remove noise. We then used the value of its derivative ($\frac{dS_{BW}}{dt}$) during model integration.

In the *α*-free model, relative changes in *FFM* to *FM* are described by the following function

$$\begin{array}{c} \frac{dFFM}{dFM}=-\frac{I-E-\rho_{FM}\frac{dBW}{dt}}{I-E-\rho_{FFM}\frac{dBW}{dt}}, \end{array}$$ (8)

which may change over time, unlike the earlier described function *α*. The calibrated parameter values of the *α*-free model were estimated in the same manner as the 2-dimensional model parameters described above. Calibrated *α*-free model parameter values are shown in S3 Table.

### Simulation and analysis

All model simulations and data fitting were performed using a commercial software package (MATLAB 2014B, MathWorks Inc., Natick, MA 2014). Both models were initialized using average rat BC measurements at the start of the study for each treatment level. Total least squares fitting was performed using functions from TLS toolbox [22].

## Results

### Evaluation of the two-dimensional rat body composition model

**Fig 1:**
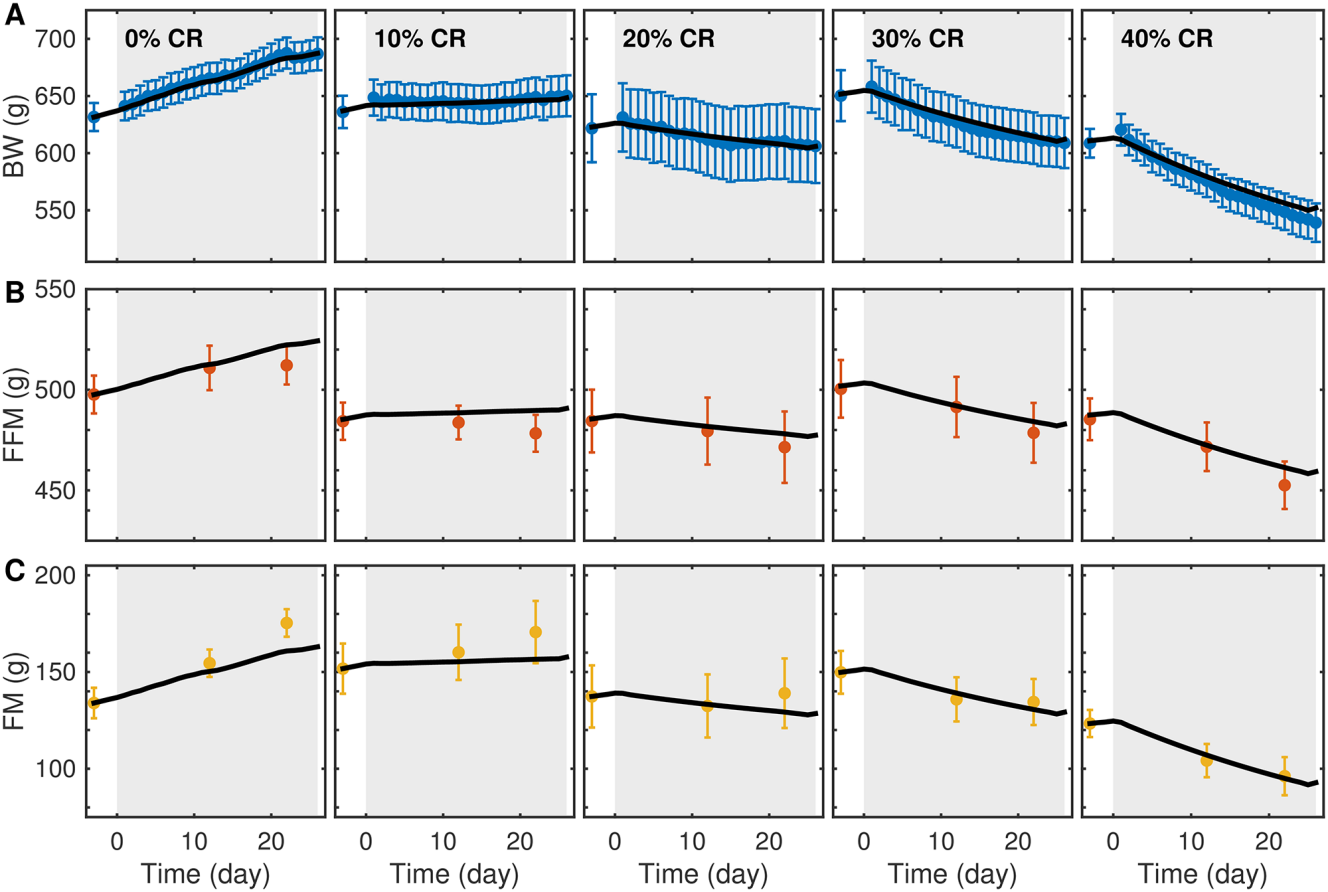
Model calibration against CR data. The calibrated model shows good agreement between model simulations (black) and experimental BW (A), FFM (B), and FM (C) measurements at all caloric restriction levels. Gray region indicates the intervention phase in each study. Error bars represent SEM (9-10 rats).

**Fig 2:**
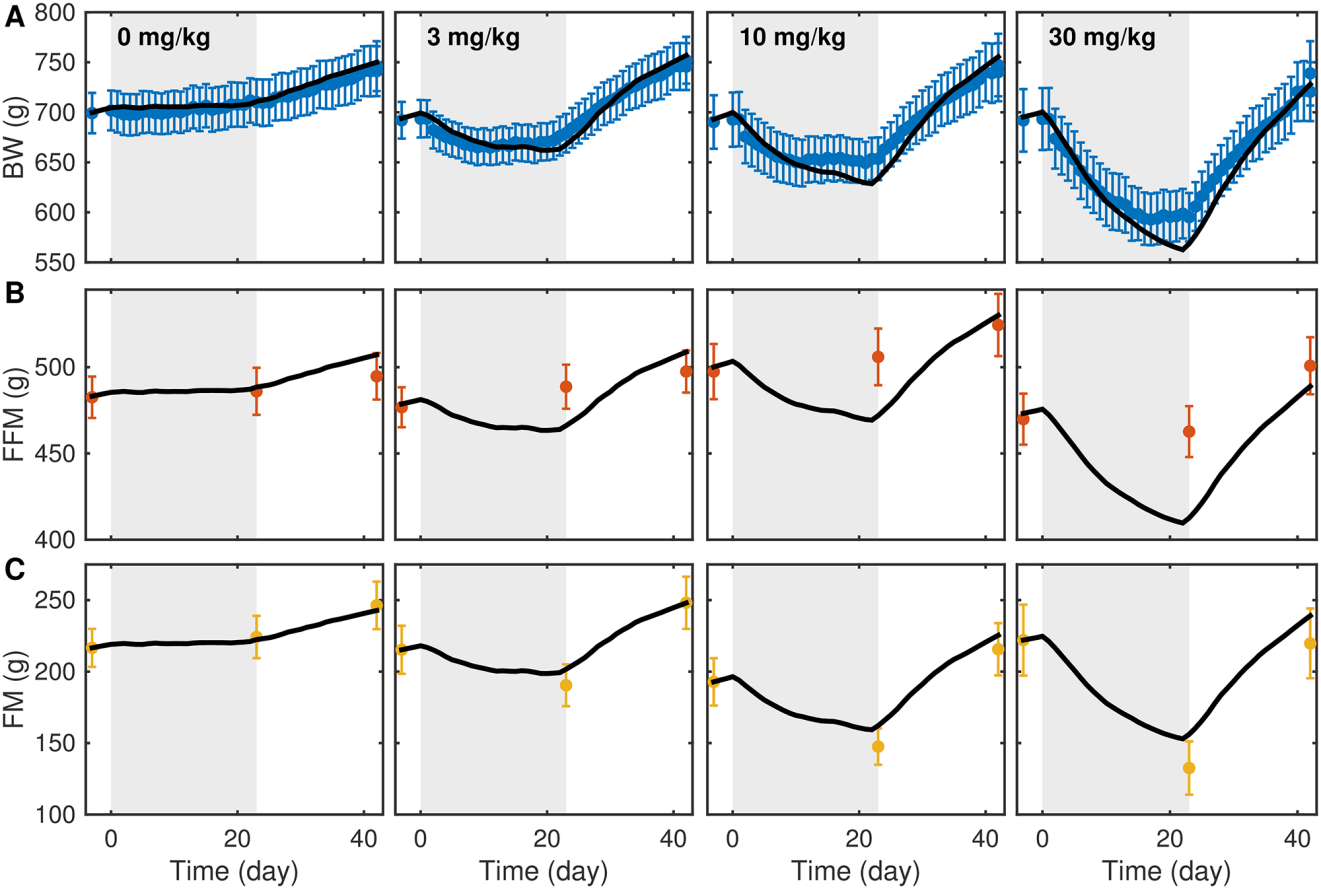
Model calibration against CB1Ra data. The calibrated model shows good agreement between model simulations (black) and experimental BW (A). The fitted model trajectories show poor agreement with FFM (B) and FM (C) measurements at the three drug dose levels. Gray region indicates the intervention phase in each study. Error bars represent SEM (9-10 rats).

The calibrated model matched longitudinal BW (Fig 1A) and BC (Fig 1B and 1C) data at all levels of CR. However, while the model matched longitudinal BW across the three CB1Ra dose levels, there was significant discrepancy between model simulations and BC data (Fig 2). Therefore, the best-fit model was not able to describe changes in BC in both studies simultaneously.

### Evaluation of the assumption for the body composition relationship

**Fig 3:**
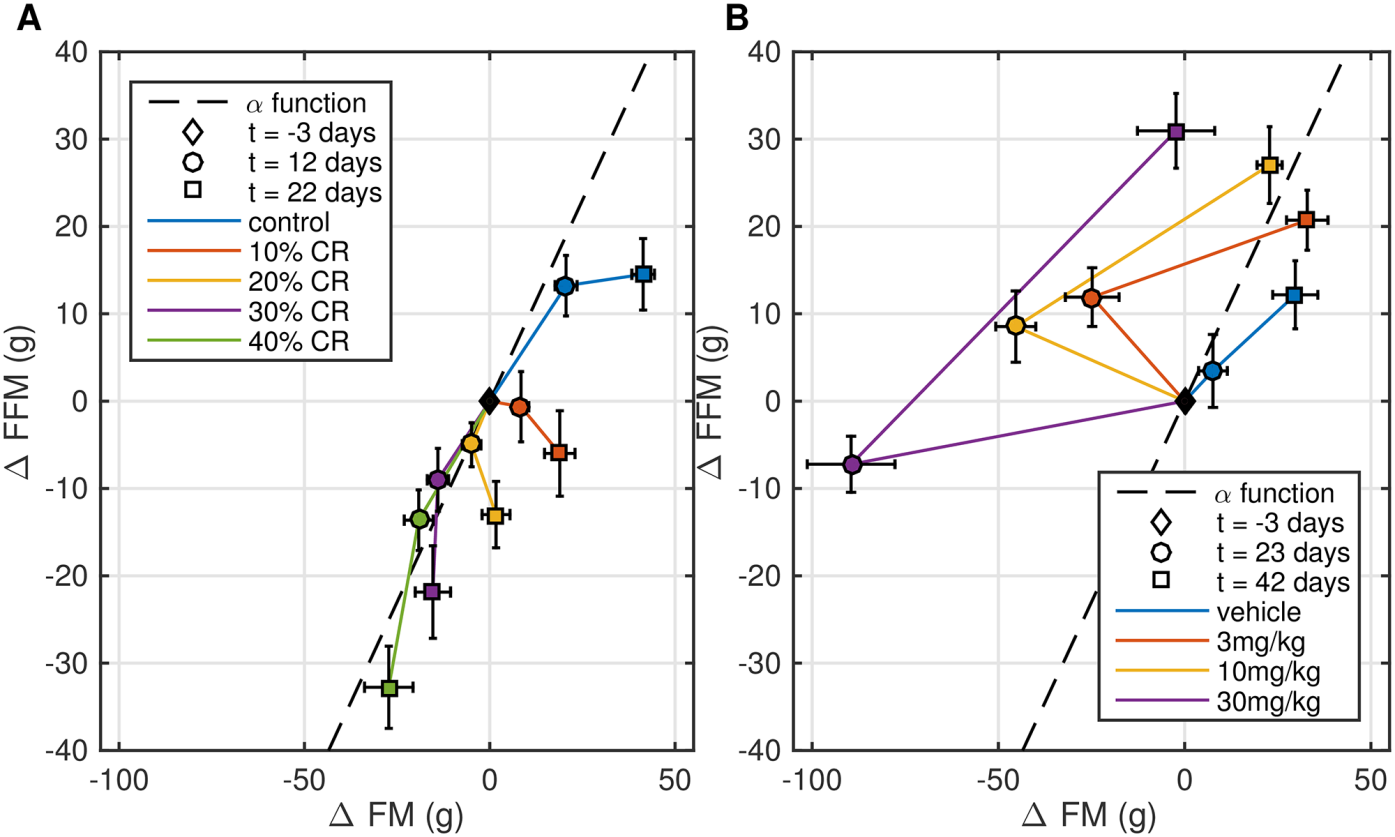
Body composition data for CR and CB1Ra treatment studies. Different color curves show changes in body composition in response to different levels of CR (A) and CB1Ra (B). Changes in FFM and FM in (A) and (B) do not appear along a single pre-defined FFM-FM curve (dashed line) defined by the energy partition function [7, 8, 21]. The different shapes correspond to three time points when BC measurements were taken. The colored line segments connecting data points are meant to guide the reader’s eye. Error bars represent SEM (9-10 rats).

A plot of longitudinal changes in BC components shows that different levels of CR produce BC trajectories that do not match a single curve defined by the constant function *α* (Fig 3A). While FFM decreases for all levels of CR, FM only decreases under significant CR conditions. In the CB1Ra study, we similarly find that different drug doses result in unique BC trajectories that do not match the function *α* assumed by the original model. Fig 3B shows that the curves for the washout and drug treatment phases of the study are not in agreement. Unlike CR treatment, CB1Ra treatment produces a dose-dependent decrease in FM, but only lowers FFM at the highest dose. We further show how the assumed unique function *α* constrains resulting model solutions (S4 and S5 Figs).

### Evaluation of the *α*-free model

**Fig 4:**
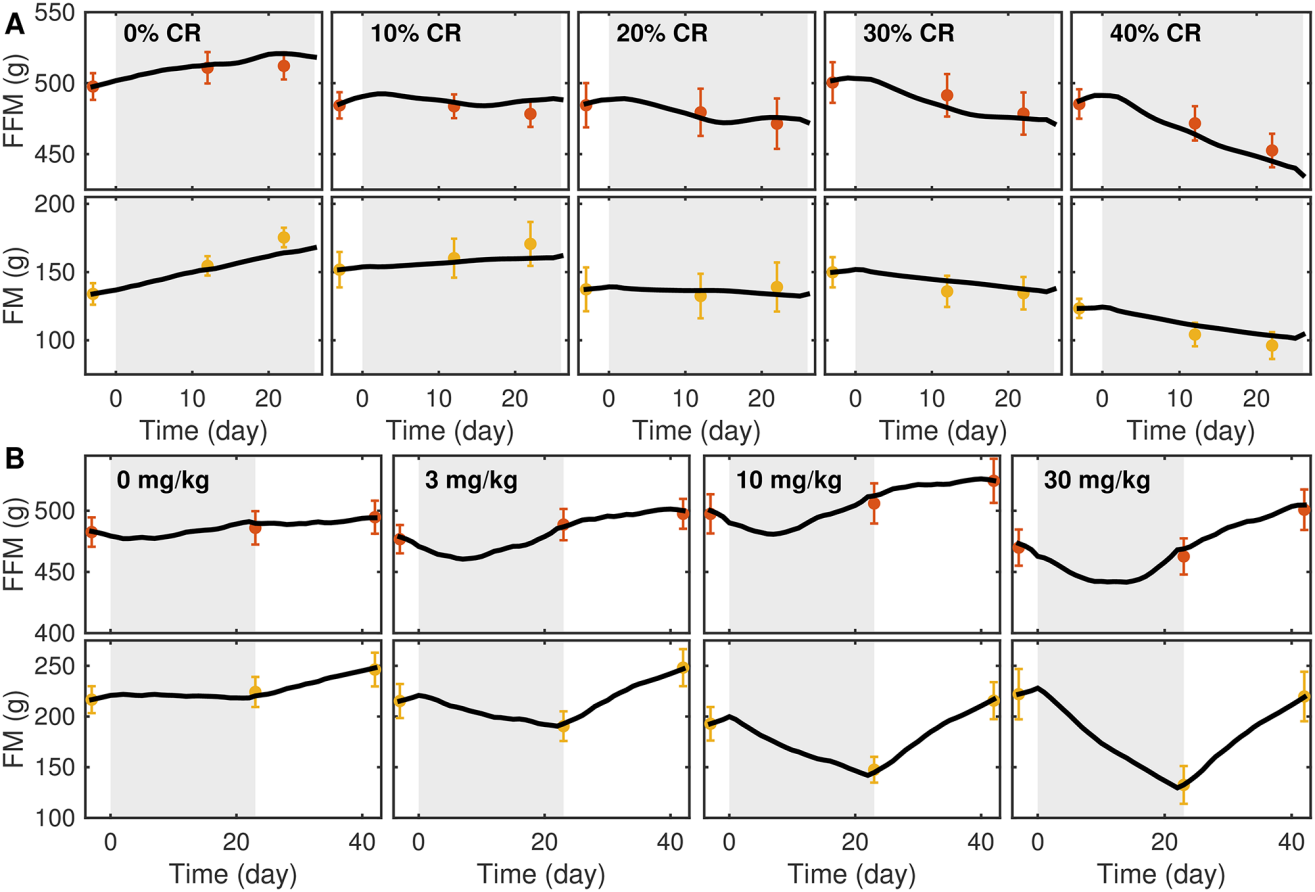
The α-free model fits BC in CR and CB1Ra BC studies. Simulations (black) of the *α*-free model show agreement with FFM (red) and FM (yellow) measurements in CR (A) and CB1Ra (B) intervention studies. Gray region indicates treatment phases in the two studies. Error bars represent SEM (9-10 rats).

**Fig 5:**
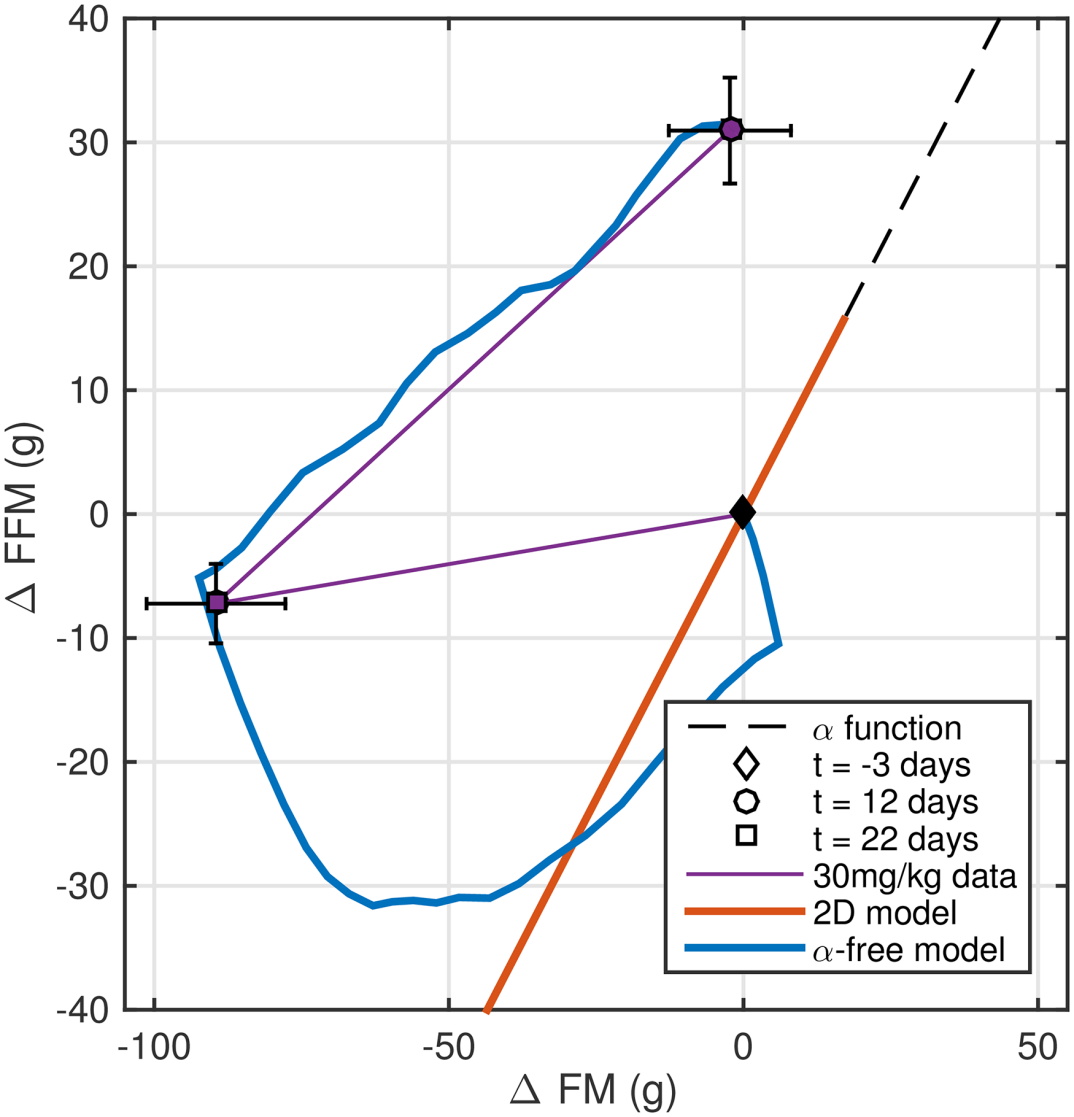
The α-free model allows for estimation of body composition changes compared to the two-dimensional model. Body composition simulations of the *α*-free model (blue) can be used to estimate FM and FFM outside of measured time points, black. The two-dimensional model simulation based on the *α* function (red) is unable to accurately capture changes in FM and FFM (purple). Only data for the 30 mg/kg dose is shown, with the purple line segments meant to guide the reader’s eye. Error bars represent SEM (9-10 rats).

The calibrated *α*-free model matched longitudinal BC data at all caloric restriction levels and drug doses (Fig 4). Fig 5 shows that the calibrated *α*-free model (blue) produced a BC trajectory that matches the drug treatment data (30 mg/kg), unlike the two-dimensional model (red) based on the constant function *α* (dashed black curve). Similar findings for other drug treatment doses are shown in S4 and S5 Figs.

Best-fit *α*-free model parameters show only slight differences from the best-fit two-dimensional model parameters (S2 and S3 Tables). Physical activity constant (*λ* = 0.087 kcal/g/day) is within the range predicted by allometric scaling of mouse physical activity values (0.07−0.12 kcal/g/day) computed in [8]. We found a non-zero basal thermogenesis for the CB1Ra study (*K*_*CB*1_ = −16.6 kcal/day). The maximum drug effect on EE was estimated to be 37 kcal/day with half-maximal drug dose around 10 mg. The parameter associated with handling stress due to drug and vehicle administration was estimated to contribute 13 kcal/day of additional EE.

## Discussion

A mathematical framework describing energy balance changes in response to therapeutic interventions can help guide a variety of drug discovery and development projects including improving study design and facilitating translation of pre-clinical findings to the clinic. While detailed, physiologically based models of human energy metabolism have been developed [6], similarly detailed models describing body weight/composition in rodent species are lacking. As an initial step toward this goal, we adapted a previously described two-dimensional energy balance model for mice [7] to SD rats. Our aim was to simultaneously describe two different, common intervention studies, thereby reducing the biases associated with fitting the model to each of the studies independently. Furthermore, the ability of the model to describe multiple types of interventions provides enhanced confidence in its predictive capabilities and expands the scope of its application.

Model parameters were adapted for known species differences and calibrated by fitting parameters describing energy partition, basal thermogenesis, physical activity, and drug-mediated energy expenditure to data from two independent rat studies. The calibrated model recapitulated CR BW and BC data (Fig 1), but failed to capture changes in BC as the result of CB1Ra treatment (Fig 2). Even though the model reproduced changes in BW in both studies, the large discrepancy with BC measurements in the latter study suggests that the model cannot describe the mechanism of weight change appropriately. Specifically, although the model may reproduce body weight data, it may not be sufficient to explain differences in body composition under different treatment paradigms and, therefore, associated changes in energy metabolism.

To address the failure of the two-dimensional model to capture CB1Ra BC data, we evaluated one of the model’s key assumptions (the *α* function), which describes the relative change in FM and FFM over time. To estimate *α*, the authors in [8] and [9] fit an exponential function to the population BC data from a long-term (> 4months) growth study in mice. Using a similar approach, we combined the data from the individual arms of our rat CR study and due to the lack of an obvious non-linear relationship between FM and FFM (S2 Fig), we chose to fit a constant *α* function to describe the CR data. The model with the constant *α* worked well to describe the CR study (Fig 1) in spite of the function’s shortcomings (Fig 3A). This is most likely due to the robustness of the two-dimensional model to *α* misspecification [23]. However, due to more significant changes in FM relative to FFM with CB1Ra treatment (Fig 3B), the constant *α* assumption limited the model’s ability to describe BC data in this case. Therefore, the direct application of a fixed FFM-FM relationship to the dynamics of short-term weight loss/regain in rats appears insufficient. It has been shown that dynamics of short-term weight loss are different from long-term studies, where application of an empirical “Forbes curve” relating FM and FFM appears to be suitable [24, 25]. As such, a mechanistic description of the effects of FI and EE on individual BC components for non-chronic studies warrants development. This has already been accomplished in a human metabolism model [6], but is yet to be done in a rodent system.

Lacking a mechanistic description for relative changes in FFM and FM, we derived an *α*-free model from energy and mass balance equations to test whether the EE function along with direct BW input could describe BC changes in the two studies. The model was able to fit both BW and BC data from the CR and CB1Ra studies simultaneously, indicating that the *α*-free model may be suitable to predict changes in BC and EE based on collected FI and BW data. The fact that the best-fit *α*-free model parameters (S3 Table) are not significantly different from the best-fit two-dimensional model parameters (S2 Table) provides further support that the constraint imposed by the function *α* was a major limitation of the two-dimensional model. While BW and FI data are measured regularly in pre-clinical studies, BC measurements require additional animal handling, which has an effect on EE that may confound study results [26]. Therefore, by using a predictive *α*-free model we may be able to eliminate the need for regular BC measurements, reducing study complexity and eliminating confounding factors.

To confirm the dose-dependent drug effect on EE predicted by the *α*-free model (S6 Fig), additional EE measurements are necessary. We propose to run the two described intervention studies within a single colony of rats inside metabolic cages, which would minimize EE differences arising from using separate animal cohorts and provide the necessary EE data to corroborate model predictions. In future studies, a direct measurement-supported EE function will allow us to use BW and FI measurements along with the *α*-free model to estimate not only changes in BC, but EE as well. Ultimately, a validated model could reduce the need for metabolic cages and associated effects on handling behavior [27].

Direct EE measurements may also help to identify other mediators of BW change that currently are implicitly captured by the EE function. In particular, since FFM is assumed to be homogeneous, which is exemplified by its constant energy density (*ρ*_*FFM*_), changes in extracellular water in short-term studies may be misinterpreted as changes in EE (S1 Text). It has been shown that a significant component of BW loss can be attributed to extracellular water loss [28, 29]. This water contribution is particularly important for short-term studies, where regain of fluid balance may not be fully achieved. In addition to the proposed use of metabolic cages, we suggest direct measurements of fluid balance for better characterization of BW regulation in short-term studies. These measurements may provide support for an explicit description of fluid disposition in future iterations of the model and potentially help identify mechanisms responsible for changes in BC. While cumbersome, these measurements would only be required for initial model evaluation, with model predictions serving to replace them in subsequent studies.

The approach of integrating disparate datasets obtained under a variety of conditions in a unifying mathematical framework presents a robust path to the development of predictive models that may obviate the need to collect very detailed measurements in the future. Our use of a simple two-dimensional energy balance model in conjunction with available data in rats illustrates how a mathematical modeling framework can help identify knowledge gaps in our understanding of energy balance and its effect on body composition. Proposed measurements of EE and fluid intake and excretion will help verify the derived *α*-free model and improve upon the model’s ability to predict changes in EE and BC in short-term studies. In addition to these measurements, further studies are required to elucidate the mechanisms responsible for the non-trivial changes in BC, which is important for improved preclinical-to-clinical translation of short-term interventional study results.

## Supporting Information

S1 Text Derivation of the two-dimensional model with water. (PDF)

S1 Fig Food intake from the two intervention studies. (A) Caloric restriction study food intake measurements. Baseline FI was established over 4 days, 12 days before the start of caloric restriction period. (B) CB1Ra treatment study food intake measurements. Baseline food intake was set equal to day 0 measurement. Gray region indicates the intervention phase in each study. Error bars represent SEM (9-10 rats).
(PDF)

S2 Fig Caloric restriction study DIO rat body composition curve. Using total least squares (TLS) regression, we fit a linear model (magenta) to the pooled CR body composition data (*FFM* = 0.92*FM* + 357). Colored circles indicate averages for each treatment group (9-10 rats each) with error bars corresponding to SEM. Gray points represent individual rat measurements.
(PDF)

S3 Fig Fitting energy expenditure model parameters. Estimators for parameters *λ* and K in the energy expenditure model show high correlation as indicated by the error function color plot. We set K = 0 and estimated optimal value for *λ* (red star).
(PDF)

S4 Fig The two-dimensional model fit to BC data for CR and CB1Ra treatment studies. Thin color curves show changes in body composition in response to different levels of CR (A) and CB1Ra (B). Thick colored curves are model simulations that follow a trajectory limited to the curve defined by function *α* (black dashed), which prevents optimal fitting of CB1Ra treatment BC data in (B). The different shapes correspond to three time points when BC measurements were taken. The dashed colored line segments connecting data points are meant to guide the reader’s eye. Error bars represent SEM (9-10 rats).
(PDF)

S5 Fig The *α*-free model fit to BC data for CR and CB1Ra treatment studies. Thin color curves show changes in body composition in response to different levels of CR (A) and CB1Ra (B). Thick colored curves are model simulations, which are not limited to the curve defined by function *α* (black dashed), allowing for optimal fitting of CR and CB1Ra treatment BC data. The different symbols correspond to three time points when BC measurements were taken. The dashed colored line segments connecting data points are meant to guide the reader’s eye. Error bars represent SEM (9-10 rats).
(PDF)

S6 Fig Predictions of *α*-free model fit to BC data. (A) Energy expenditure predicted by the *α*-free model fit to BC data. (B) Rat handling stress (vehicle) plus dose-dependent drug effects on energy expenditure predicted by the *α*-free model fit to BC data. Colors in (A) correspond to the legend in (B).
(PDF)

S1 Table Translated parameter values of the 2-dimensional model. (PDF)

S2 Table Calibrated parameter values of the 2-dimensional model. (PDF)

S3 Table Calibrated parameter values of the *α*-free model. (PDF)

S1 Supporting Dataset Individual rat body weight, fat mass, food intake, and rimonabant plasma concentration measurements. (XLSX)

S1 Code Matlab scripts and functions used to generate the main figures and simulate the model. (ZIP)

## References

[1] Williams EP, Mesidor M, Winters K, Dubbert PM, Wyatt SB. Overweight and Obesity: Prevalence, Consequences, and Causes of a Growing Public Health Problem. Current Obesity Reports. 2015;4(3):363–370. 10.1007/s13679-015-0169-4 26627494

[2] Wu T, Gao X, Chen M, Van Dam R. Long-term effectiveness of diet-plus-exercise interventions vs. diet-only interventions for weight loss: a meta-analysis. Obesity reviews. 2009;10(3):313–323. 10.1111/j.1467-789X.2008.00547.x 19175510

[3] Smoliga JM, Vang O, Baur JA. Challenges of translating basic research into therapeutics: resveratrol as an example. The Journals of Gerontology Series A: Biological Sciences and Medical Sciences. 2012;67(2):158–167. 10.1093/gerona/glr062 PMC3261440 21746739

[4] Rodgers RJ, Tschöp MH, Wilding JP. Anti-obesity drugs: past, present and future. Disease models & mechanisms. 2012;5(5):621–626. 10.1242/dmm.009621 22915024 PMC3424459

[5] Valsamakis G, Lois K, Kumar S, Mastorakos G. New molecular targets in the pathophysiology of obesity and available treatment options under investigation. Clinical obesity. 2014;4(4):209–219. 25826792 10.1111/cob.12064

[6] Hall KD. Predicting metabolic adaptation, body weight change, and energy intake in humans. American Journal of Physiology-Endocrinology and Metabolism. 2010;298(3):E449–E466. 10.1152/ajpendo.00559.2009 19934407 PMC2838532

[7] Guo J, Hall KD. Estimating the continuous-time dynamics of energy and fat metabolism in mice. PLoS computational biology. 2009;5(9):e1000511 10.1371/journal.pcbi.1000511 19763167 PMC2731929

[8] Guo J, Hall KD. Predicting changes of body weight, body fat, energy expenditure and metabolic fuel selection in C57BL/6 mice. PLoS One. 2011;6(1):e15961 10.1371/journal.pone.0015961 21246038 PMC3016341

[9] Gennemark P, Jansson-Löfmark R, Hyberg G, Wigstrand M, Kakol-Palm D, Håkansson P, et al A modeling approach for compounds affecting body composition. Journal of pharmacokinetics and pharmacodynamics. 2013;40(6):651–667. 10.1007/s10928-013-9337-x 24158456

[10] Gennemark P, Hjorth S, Gabrielsson J. Modeling energy intake by adding homeostatic feedback and drug intervention. Journal of pharmacokinetics and pharmacodynamics. 2015;42(1):79–96. 10.1007/s10928-014-9399-4 25388764

[11] Jacquier M, Crauste F, Soulage CO, Soula HA. A Predictive Model of the Dynamics of Body Weight and Food Intake in Rats Submitted to Caloric Restrictions. PLoS ONE. 2014 6;9(6):e100073 10.1371/journal.pone.0100073 24932616 PMC4059745

[12] Sam AH, Salem V, Ghatei MA Rimonabant: from RIO to ban. Journal of Obesity. 2011 5;2011:ID432607 10.1155/2011/432607 PMC3136184 21773005

[13] Christensen R, Kristensen PK, Bartels EM, Bliddal H, Astrup A. Efficacy and safety of the weight-loss drug rimonabant: a meta-analysis of randomised trials. The Lancet. 2007;370(9600):1706–1713. 10.1016/S0140-6736(07)61721-8 18022033

[14] Colombo G, Agabio R, Diaz G, Lobina C, Reali R, Gessa GL. Appetite suppression and weight loss after the cannabinoid antagonist SR 141716. Life sciences. 1998;63(8):PL113–PL117. 10.1016/S0024-3205(98)00322-1 9718088

[15] Thornton-Jones ZD, Kennett GA, Benwell KR, Revell DF, Misra A, Sellwood DM, et al The cannabinoid CB1 receptor inverse agonist, rimonabant, modifies body weight and adiponectin function in diet-induced obese rats as a consequence of reduced food intake. Pharmacology Biochemistry and Behavior. 2006 6;84(2):353–359. 10.1016/j.pbb.2006.06.001 16814374

[16] Poirier B, Bidouard JP, Cadrouvele C, Marniquet X, Staels B, O’connor S, et al The anti-obesity effect of rimonabant is associated with an improved serum lipid profile. Diabetes, Obesity and Metabolism. 2005;7(1):65–72. 10.1111/j.1463-1326.2004.00374.x 15642077

[17] Doyon C, Denis RG, Baraboi ED, Samson P, Lalonde J, Deshaies Y, et al Effects of rimonabant (SR141716) on fasting-induced hypothalamic-pituitary-adrenal axis and neuronal activation in lean and obese Zucker rats. Diabetes. 2006;55(12):3403–3410. 10.2337/db06-0504 17130486

[18] Herling AW, Kilp S, Elvert R, Haschke G, Kramer W. Increased energy expenditure contributes more to the body weight-reducing effect of rimonabant than reduced food intake in candy-fed wistar rats. Endocrinology. 2008;149(5):2557–2566. 10.1210/en.2007-1515 18276749

[19] Abstracts of the 2nd Asian Pacific Regional ISSX Meeting (May 11–13, 2008). Drug Metabolism Reviews. 2008;40:Sup2:1–226. 10.1080/03602530802110389 18600495

[20] Chow CC, Hall KD. The dynamics of human body weight change. PLoS Comput Biol. 2008;4(3):e1000045 10.1371/journal.pcbi.1000045 18369435 PMC2266991

[21] Hall KD. Mathematical modeling of energy expenditure during tissue deposition. British journal of nutrition. 2010;104(01):4–7. 10.1017/S0007114510000206 20132585

[22] Petráš I, Bednárová D. Total least squares approach to modeling: a Matlab toolbox. Acta Montanistica Slovaca. 2010;15(2):158.

[23] Thompson WC, Zhou Y, Tulukdar S, Musante CJ. PF-05231023, a Long-Acting FGF21 Analogue, Decreases Body Weight by Reduction of Food Intake in Non-Human Primates. submitted 2016. 10.1007/s10928-016-9481-1 PMC4954843 27405817

[24] Heymsfield S, Gonzalez M, Shen W, Redman L, Thomas D. Weight loss composition is one-fourth fat-free mass: a critical review and critique of this widely cited rule. Obesity Reviews. 2014;15(4):310–321. 10.1111/obr.12143 24447775 PMC3970209

[25] Forbes GB. Lean Body Mass-Body Fat Interrelationships in Humans. Nutrition reviews. 1987;45(10):225–231. 10.1111/j.1753-4887.1987.tb02684.x 3306482

[26] Careau V, Thomas D, Humphries M, Réale D. Energy metabolism and animal personality. Oikos. 2008;117(5):641–653. 10.1111/j.0030-1299.2008.16513.x

[27] Kalliokoski O, Jacobsen KR, Darusman HS, Henriksen T, Weimann A, Poulsen HE, et al Mice do not habituate to metabolism cage housing—a three week study of male BALB/c mice. PloS one. 2013;8(3):e58460 10.1371/journal.pone.0058460 23505511 PMC3591308

[28] Dicker S. Changes in the extracellular and intracellular fluid phases of muscle during starvation and dehydration in adult rats. Biochemical Journal. 1949;44(3):274 10.1042/bj0440274 16748515 PMC1274855

[29] Morrison S, Mackay C, Hurlbrink E, Wier JK, Nick MS, Millar FK. The water exchange and polyuria of rats deprived of food. Quarterly Journal of Experimental Physiology and Cognate Medical Sciences. 1967;52(1):51–67. 10.1113/expphysiol.1967.sp001885

