# Supplementary material for: Evaluation of a Mathematical Model of Rat Body Weight Regulation in Application to Caloric Restriction and Drug Treatment Studies
Source: PLoS One. 2016 May 26;11(5):e0155674. doi: 10.1371/journal.pone.0155674 (PMC4882007; doi:10.1371/journal.pone.0155674)
Supplement: S1 Text — (PDF) [file pone.0155674.s001.pdf]

Derivation of two-state model with extracellular water

In agreement with Guo and Hall (2009), we assume that fat-free mass (FFM) is primarily composed of protein (P) and water. We define lean mass (LM) composed of protein and intracellular water that hydrates it, which is characterized by protein hydration fraction $h_P = 1.6$. Extracellular water (W) is the remainder of the water, which is independent of energy-carrying components of body weight (BW). Overall mass balance is defined by the following 3 equations:

$$BW = FFM + FM \tag{1}$$
$$FFM = LM + W \tag{2}$$
$$LM = (1 + h_P)P. \tag{3}$$

Given that protein is the only energy carrying component of fat-free mass, let us derive the energy balance equation in terms of FFM using above formulation. We write down the energy balance equation for the energy-carrying components (P, FM):

$$\rho_{FM}\frac{dFM}{dt} + \rho_P\frac{dP}{dt} = I - \hat{E} \tag{4}$$

where $\hat{E}$ is true energy expenditure, I is the food intake energy, and $\rho_P = 4.7$ is the energy density of protein. Plugging Eq. 3 into Eq. 2 and taking derivative with respect to time, we get

$$\frac{dP}{dt} = \frac{1}{1 + h_P}\left(\frac{dFFM}{dt} - \frac{dW}{dt}\right) \tag{5}$$

which we then plug into Eq. 4 for $\frac{dP}{dt}$ to get

$$\rho_{FM}\frac{dFM}{dt} + \rho_{FFM}\left(\frac{dFFM}{dt} - \frac{dW}{dt}\right) = I - \hat{E} \tag{6}$$

where $\rho_{FFM} = \frac{\rho_P}{1+h_P}$ is equal to the value of energy density of fat-free mass defined in Table S1. Rearranging the terms, we obtain the following familiar energy balance equation (Main Text Eq. 6):

$$\rho_{FM}\frac{dFM}{dt} + \rho_{FFM}\frac{dFFM}{dt} = I - E \tag{7}$$

where $E = \hat{E} - \rho_{FFM}\frac{dW}{dt}$ shows that energy expenditure E includes changes in the extracellular water W in addition to the true energy expenditure $\hat{E}$. This derivation shows the need to measure body water content to differentiate between changes in BW that are driven by energy expenditure effects versus changes in body water.
