# Supplementary figures and images for: Evaluation of a Mathematical Model of Rat Body Weight Regulation in Application to Caloric Restriction and Drug Treatment Studies
Source: PLoS One. 2016 May 26;11(5):e0155674. doi: 10.1371/journal.pone.0155674 (PMC4882007; doi:10.1371/journal.pone.0155674)

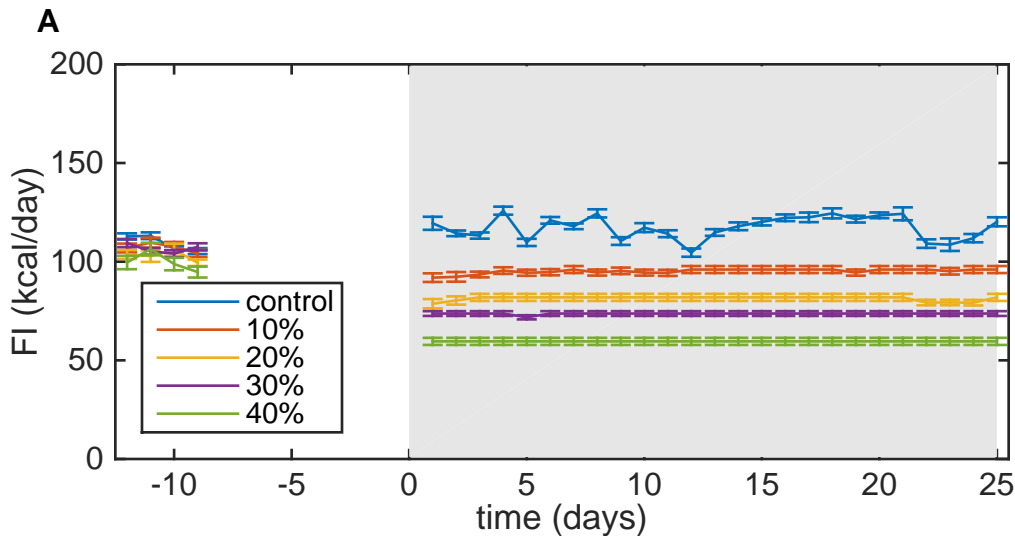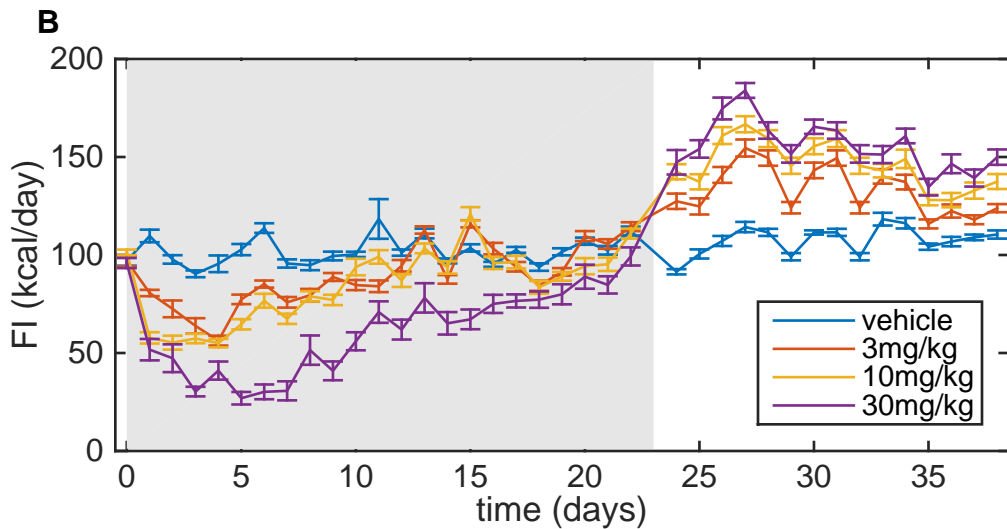

Supplement: S1 Fig — (A) Caloric restriction study food intake measurements. Baseline FI was established over 4 days, 12 days before the start of caloric restriction period. (B) CB1Ra treatment study food intake measurements. Baseline food intake was set equal to day 0 measurement. Gray region indicates the intervention phase in each study. Error bars represent SEM (9-10 rats). (PDF) [file pone.0155674.s002.pdf]

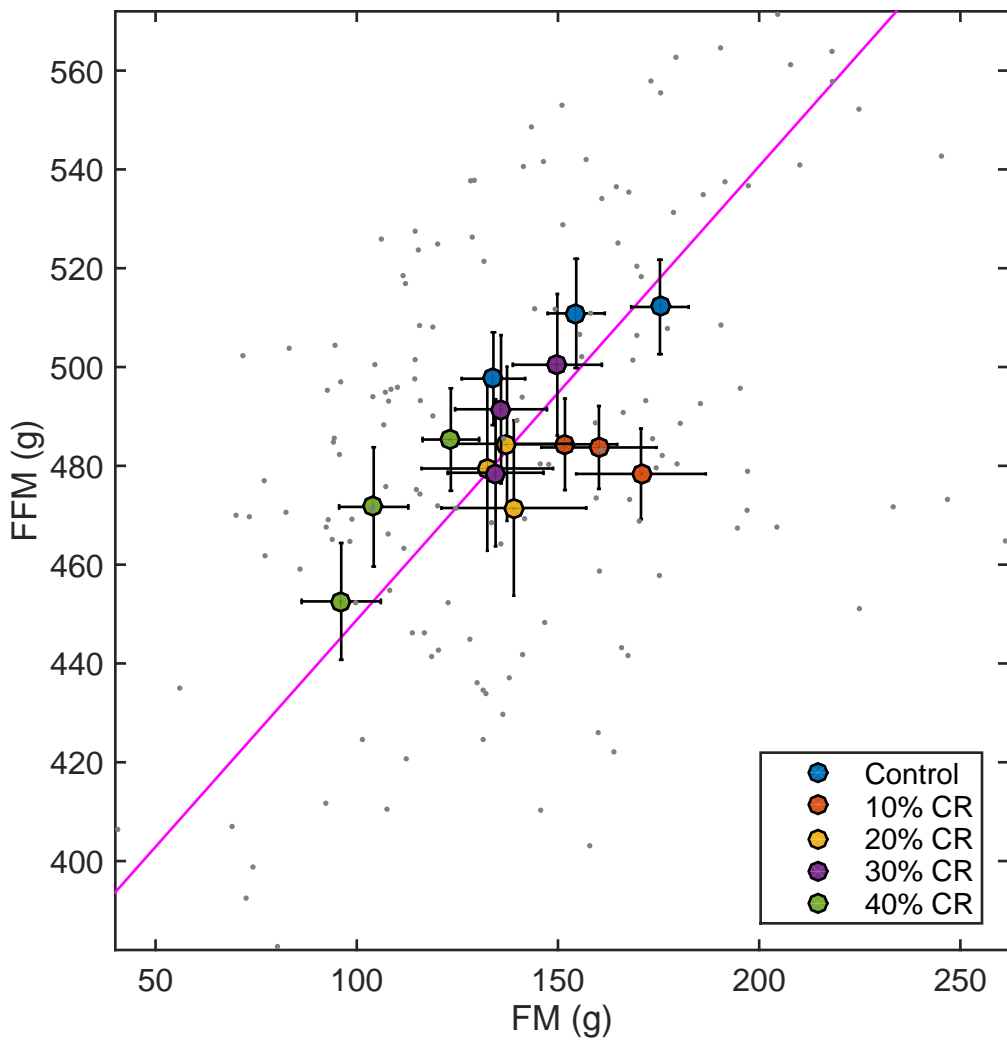

Supplement: S2 Fig — Using total least squares (TLS) regression, we fit a linear model (magenta) to the pooled CR body composition data (FFM = 0.92FM + 357). Colored circles indicate averages for each treatment group (9-10 rats each) with error bars corresponding to SEM. Gray points represent individual rat measurements. (PDF) [file pone.0155674.s003.pdf]

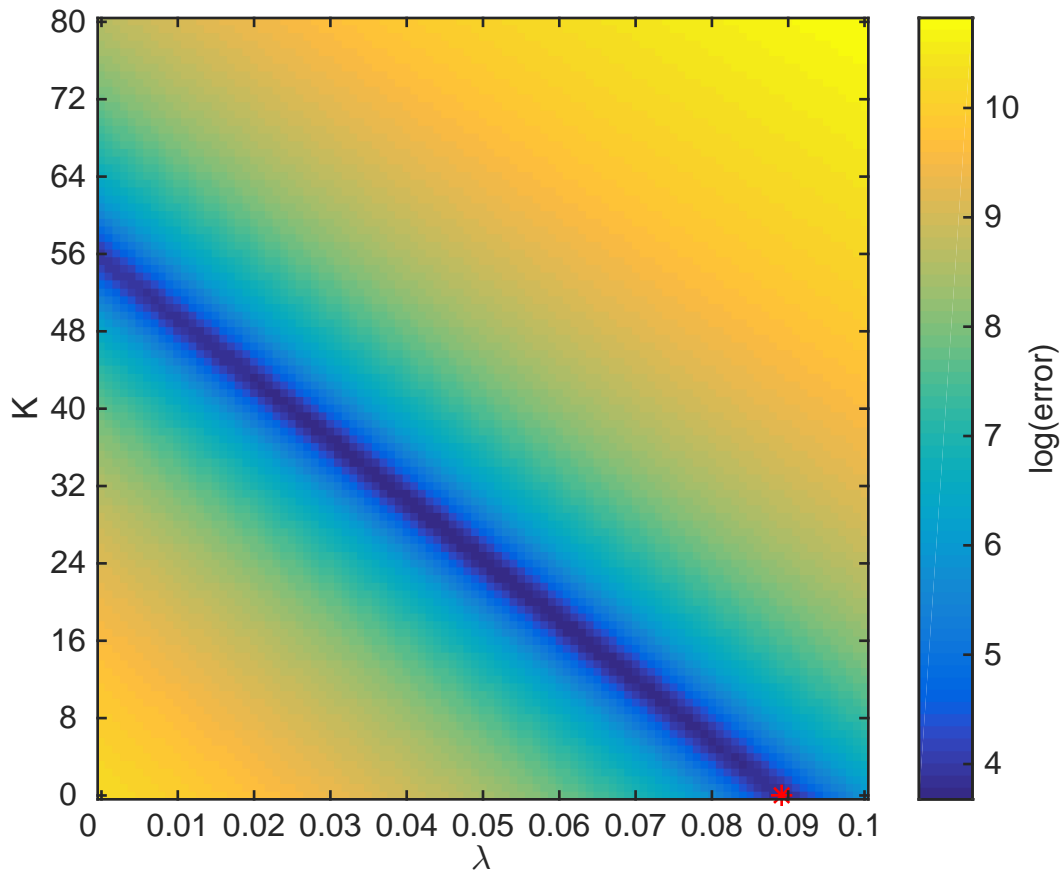

Supplement: S3 Fig — Estimators for parameters λ and K in the energy expenditure model show high correlation as indicated by the error function color plot. We set K = 0 and estimated optimal value for λ (red star). (PDF) [file pone.0155674.s004.pdf]

**A**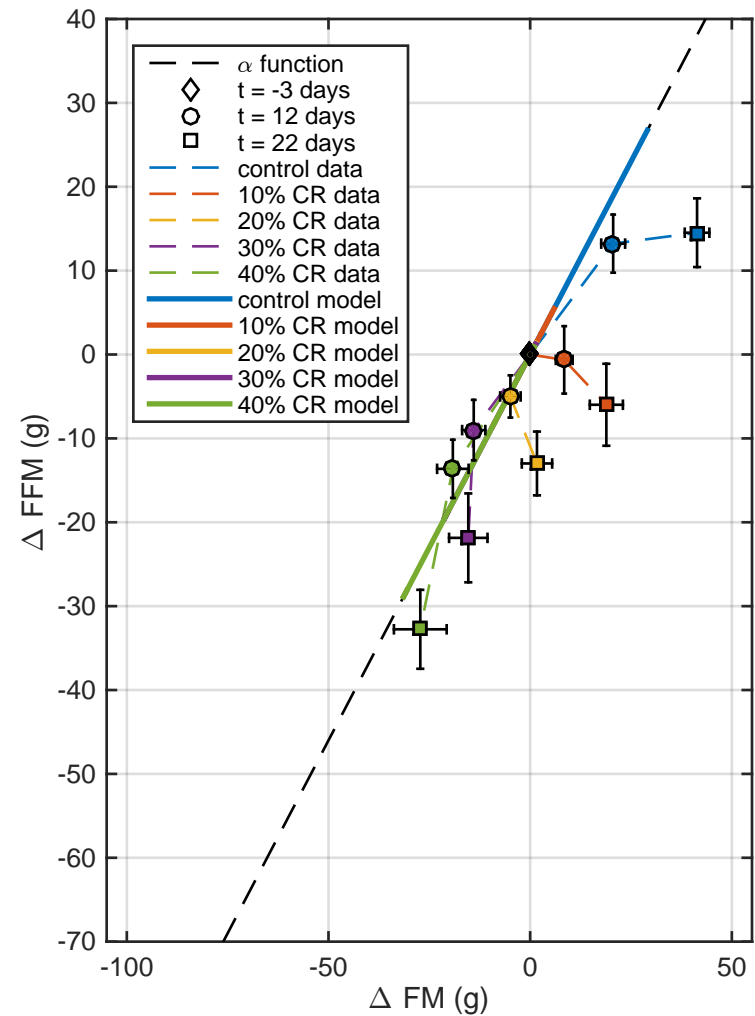**B**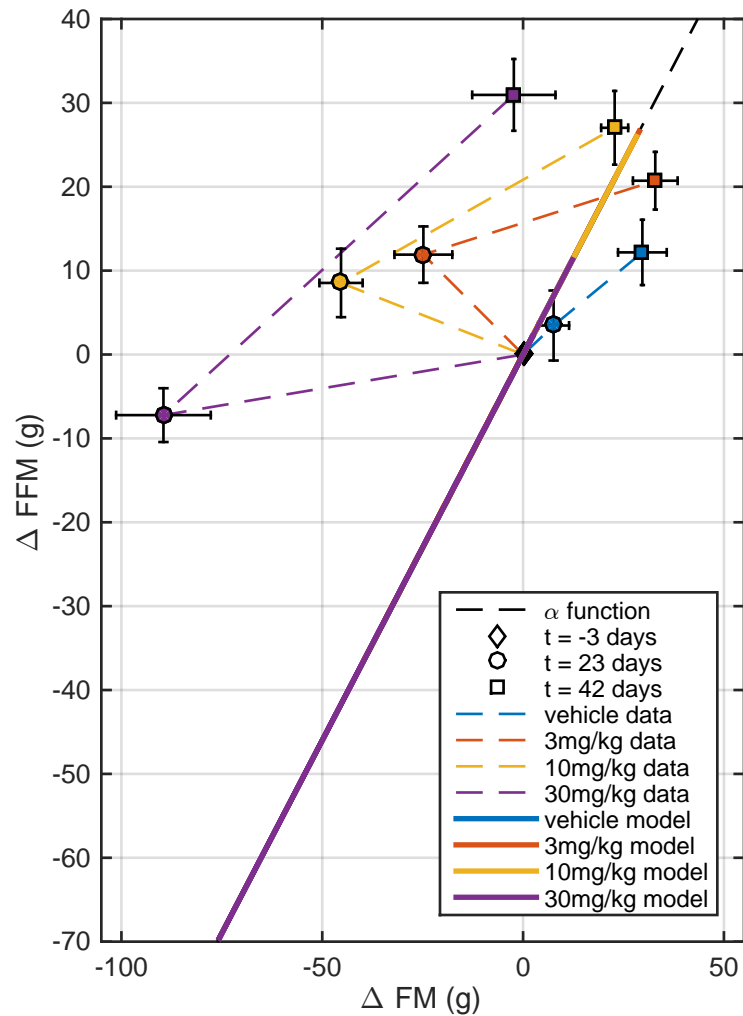

Supplement: S4 Fig — Thin color curves show changes in body composition in response to different levels of CR (A) and CB1Ra (B). Thick colored curves are model simulations that follow a trajectory limited to the curve defined by function α (black dashed), which prevents optimal fitting of CB1Ra treatment BC data in (B). The different shapes correspond to three time points when BC measurements were taken. The dashed colored line segments connecting data points are meant to guide the reader’s eye. Error bars represent SEM (9-10 rats). (PDF) [file pone.0155674.s005.pdf]

**A**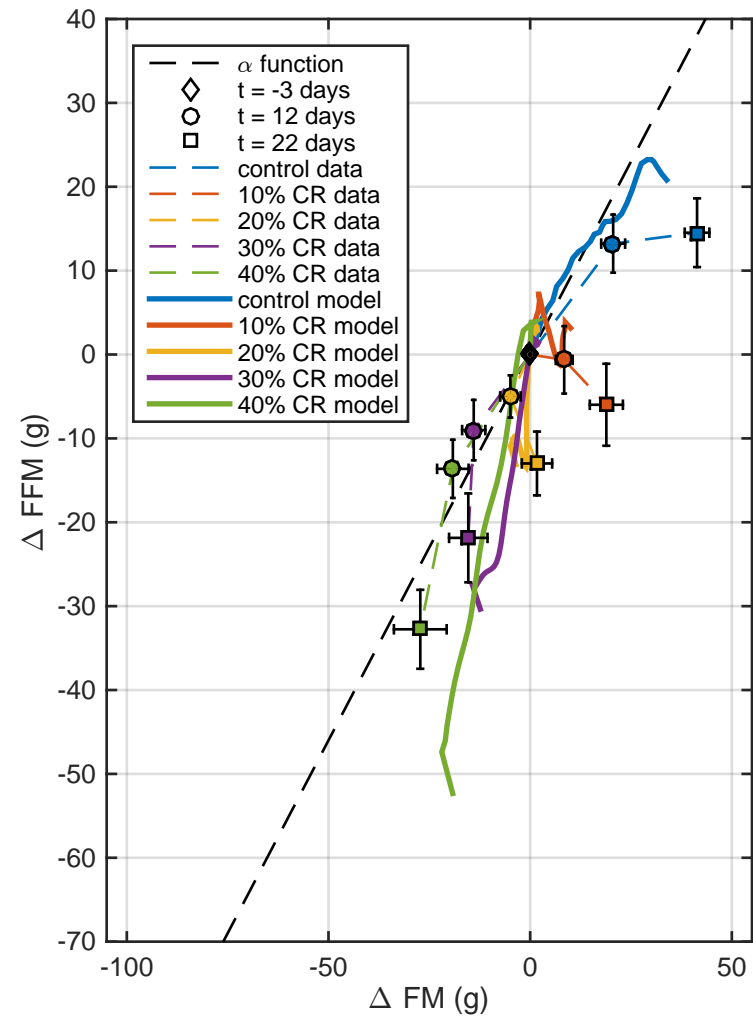**B**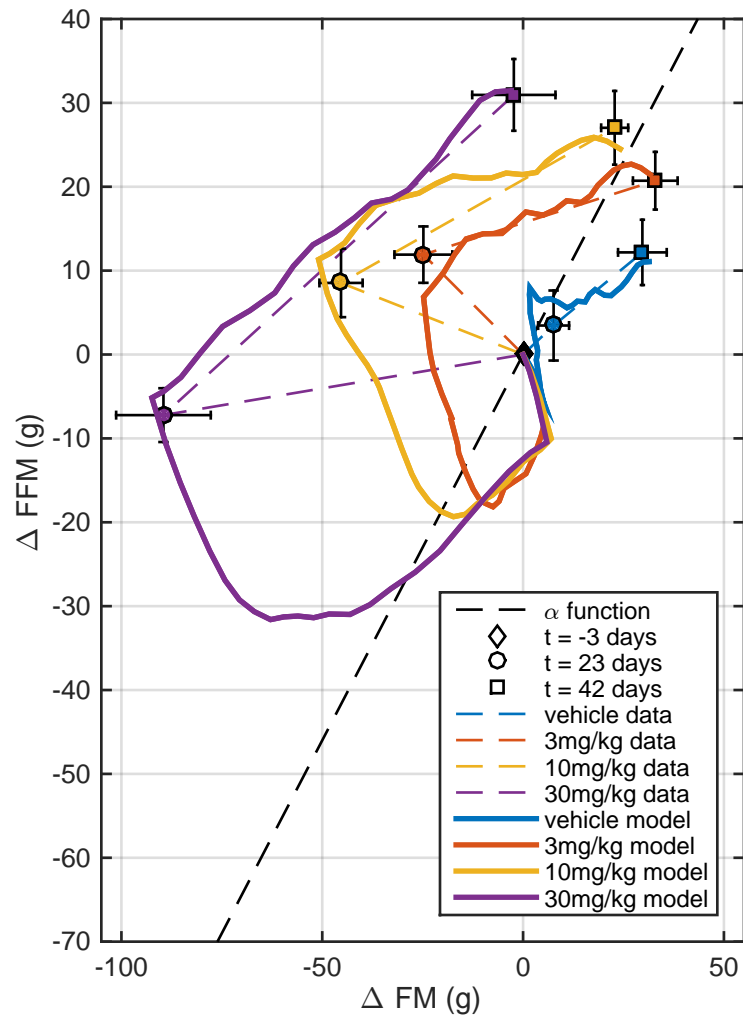

Supplement: S5 Fig — Thin color curves show changes in body composition in response to different levels of CR (A) and CB1Ra (B). Thick colored curves are model simulations, which are not limited to the curve defined by function α (black dashed), allowing for optimal fitting of CR and CB1Ra treatment BC data. The different symbols correspond to three time points when BC measurements were taken. The dashed colored line segments connecting data points are meant to guide the reader’s eye. Error bars represent SEM (9-10 rats). (PDF) [file pone.0155674.s006.pdf]

**A**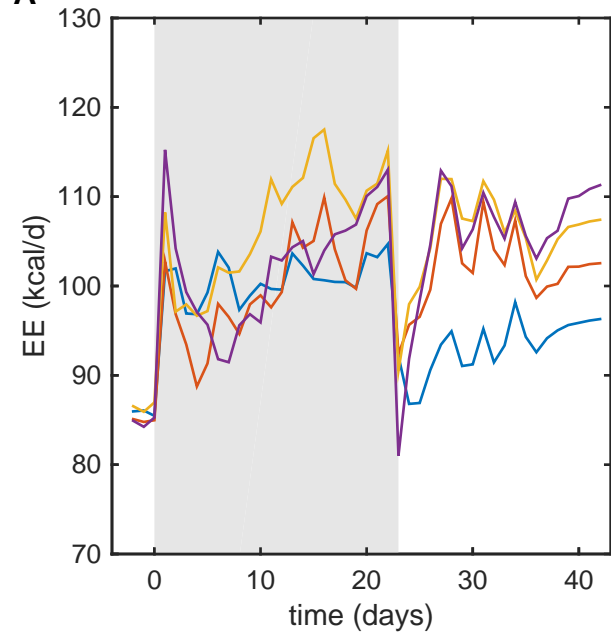**B**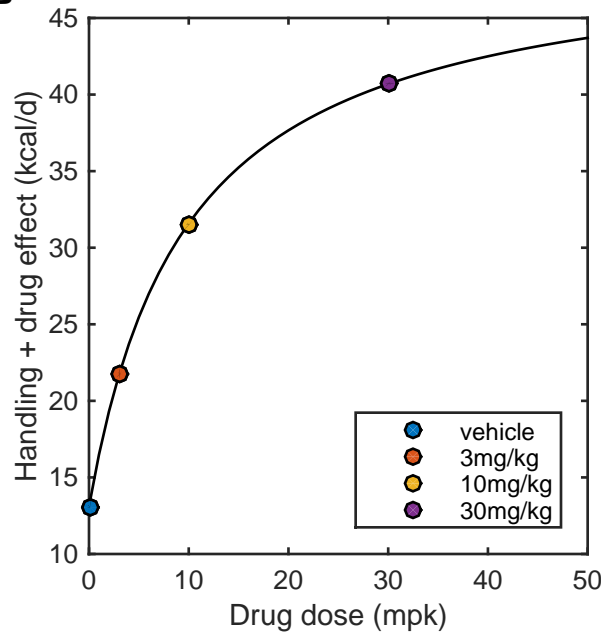

Supplement: S6 Fig — (A) Energy expenditure predicted by the α-free model fit to BC data. (B) Rat handling stress (vehicle) plus dose-dependent drug effects on energy expenditure predicted by the α-free model fit to BC data. Colors in (A) correspond to the legend in (B). (PDF) [file pone.0155674.s007.pdf]
