# Supplementary material for: Evaluation of a Mathematical Model of Rat Body Weight Regulation in Application to Caloric Restriction and Drug Treatment Studies
Source: PLoS One. 2016 May 26;11(5):e0155674. doi: 10.1371/journal.pone.0155674 (PMC4882007; doi:10.1371/journal.pone.0155674)
Supplement: S1 Table — (PDF) [file pone.0155674.s008.pdf]

Translated parameter values of the 2-dimensional model.

Parameter	Description	Value	Units	Reference
ρ_{FM}	Fat mass (FM) energy density	9.4	kcal/g	Guo and Hall (2011)
ρ_{FFM}	Fat-free mass (FFM) energy density	1.8	kcal/g	Guo and Hall (2011)
γ_{FM}	FM metabolic rate	0.012	kcal/g/day	this work
γ_{FFM}	FFM metabolic rate	0.081	kcal/g/day	this work
η_{FM}	FM deposition cost	0.18	kcal/g/day	Guo and Hall (2011)
η_{FFM}	FFM deposition cost	0.23	kcal/g/day	Guo and Hall (2011)
β	Diet-induced thermogenesis	0.4	unitless	Hall (2010)
