# Supplementary material for: Evaluation of a Mathematical Model of Rat Body Weight Regulation in Application to Caloric Restriction and Drug Treatment Studies
Source: PLoS One. 2016 May 26;11(5):e0155674. doi: 10.1371/journal.pone.0155674 (PMC4882007; doi:10.1371/journal.pone.0155674)
Supplement: S2 Table — (PDF) [file pone.0155674.s009.pdf]

**Calibrated parameter values of the 2-dimensional model.**

| Parameter | Description | Value | Units |
|---|---|---|---|
| $\alpha$ | slope of energy partition function | 0.92 | unitless |
| $\lambda$ | Physical activity constant | 0.089 | kcal/g/day |
| $K_{CR}$ | Basal thermogenesis | 0 | kcal/d |
| $K_{CB1}$ | Energy imbalance during CB1 treatment | -16.9 | kcal/d |
| $D_0$ | Handling stress energy expenditure effect | 10.3 | kcal/d |
| $D_{max}$ | Maximum drug energy expenditure effect | 34.8 | kcal/d |
| $ED_{50}$ | Half-maximal effect drug dose | 9.8 | mg/kg |
