# Supplementary material for: Evaluation of a Mathematical Model of Rat Body Weight Regulation in Application to Caloric Restriction and Drug Treatment Studies
Source: PLoS One. 2016 May 26;11(5):e0155674. doi: 10.1371/journal.pone.0155674 (PMC4882007; doi:10.1371/journal.pone.0155674)
Supplement: S3 Table — (PDF) [file pone.0155674.s010.pdf]

Calibrated parameter values of the α-free model.

Parameter	Description	Value	Units
λ	Physical activity constant	0.087	kcal/g/day
K_{CR}	Basal thermogenesis	0	kcal/d
K_{CB1}	Energy imbalance during CB1 treatment	-16.6	kcal/d
D_0	Handling stress energy expenditure effect	13.1	kcal/d
D_{max}	Maximum drug energy expenditure effect	36.6	kcal/d
ED_{50}	Half-maximal effect drug dose	9.8	mg/kg
