# Supplementary material for: Evaluation of a Mathematical Model of Rat Body Weight Regulation in Application to Caloric Restriction and Drug Treatment Studies
Source: PLoS One. 2016 May 26;11(5):e0155674. doi: 10.1371/journal.pone.0155674 (PMC4882007; doi:10.1371/journal.pone.0155674)
Supplement: S1 Code — (ZIP) [file pone.0155674.s012.zip › MATLAB code/README.rtf]

README describes files included in this folder. These files were tested in MATLAB v 2014b (MathWorks, Natick, MA). The code also uses statistics toolbox.generate_figures.m - this script generates all of the main text figures and most of the supplementary figures (it call the other matlab files in this folder).FigXX.m - these functions generate specified figuresload-data.m - this function is called to load data contained in cr_rat_bw_fi_bc_data.mat and rimonabant_rat_bw_fi_bc_data.mat files. plotRatExpData.m - this function is called to plot original study data.run_BWFIM.m - this function integrates rhs_BWFIM.m using ode45 with specified parameters. rhs_BWFIM.m - this function contains the right hand side equations of the two-dimensional model of FM and FFM.herrorbar.m - this function is used in plotting of horizontal error bars. It was obtained from MATLAB Central File Exchange, and is owned by Jos van der Geest (jos@jasen.nl).fit_2D_data.m - this function calculates best fit line based on input data points using total least squares algorithm. It was obtained from MTALAB Central File Exchange and is described in [1].References1. Ivo Petras and Dagmar Bednarova Total Least Squares Approach to Modeling: A Matlab Toolbox, Acta Montanistica Slovaca, vol. 15, no. 2, 2010, pp. 158-170. 
